# Novel molecular marker-assisted strategy for production of wheat–*Leymus mollis* chromosome addition lines

**DOI:** 10.1038/s41598-018-34545-x

**Published:** 2018-10-31

**Authors:** Offiong U. Edet, Yasir S. A. Gorafi, Seong-woo Cho, Masahiro Kishii, Hisashi Tsujimoto

**Affiliations:** 1 Arid Land Research Center, Tottori University, Tottori, Japan; 2 United Graduate School of Agricultural Sciences, Tottori University, Tottori, Japan; 3 Agricultural Research Corporation (ARC), Wad Madani, Sudan; 4 Department of Crop Science and Biotechnology, Chonbuk National University, Jeonju, Republic of Korea; 5 International Maize and Wheat Improvement Center (CIMMYT), El Batan, Mexico

## Abstract

Developing wheat–alien chromosome introgression lines to improve bread wheat’s resistance to stresses, such as drought, salinity stress and diseases, requires reliable markers to identify and characterize the alien chromatins. *Leymus mollis* is a wild relative of bread wheat resistant to salinity and economically important diseases of wheat, but its genome sequence and cytological markers are not available. We devised a molecular marker-assisted strategy for *L. mollis* chromosome identification and applied it to produce 10 wheat–*L. mollis* chromosome addition lines. Using 47 *L. racemosus* genome polymorphic PCR markers and DArTseq genotyping, we distinguished the *L. mollis* chromosomes and differentiated disomic and monosomic lines by progeny test. DArTseq genotyping generated 14,530 *L. mollis* SNP markers and the chromosome-specific SNP markers were used to determine the homoeologous groups of *L. mollis* chromosomes in the addition lines. To validate the marker-based results, genomic *in situ* hybridization was applied to confirm the presence and cytological status of *L. mollis* chromosomes in the lines. This study demonstrates that adequate molecular markers allow the production and characterization of wheat–alien addition lines without *in situ* hybridization, which saves considerable time and effort.

## Introduction

*Leymus* species are prominent in Triticeae studies as pasture plants and gene sources for improving bread wheat^1–6^. They are reported to have the Ns genomes from *Psathyrostachys* and Xm genomes from an unknown source^5,7,8^. However, recent molecular analyses indicate that both genomes are from *Psathyrostachys* and that tetraploid *Leymus* species are segmental polyploids (2n = 4x = 28, Ns_1_Ns_1_Ns_2_Ns_2_)^4,9^. This genomic representation of tetraploid *Leymus* species as segmental polyploids is consistently used in this article.

Reports show that *Leymus* species are resistant to salinity and economically important wheat diseases^6,10,11^. Their chromosome segments in bread wheat genetic background improve wheat’s tolerance to biotic and abiotic stresses, such as *Fusarium* head blight, stripe rust and powdery mildew diseases^3,6,12–17^, heat stress^18^, aluminium toxicity^19^, and salinity stress^20^. The recognition of the potentials of *Leymus* species as valuable gene sources for the improvement of wheat dates back to the 1960s when Tsitsin reported the production of different combinations of wheat–*Leymus* amphidiploids^21^. Subsequent studies in this direction have demonstrated high cytogenetic stability in wheat–*L. mollis* octoploids and varying segregation and transmission rates of alien (*L. mollis*) chromosomes in different backcross generations of wheat–*L. mollis* backcross populations^22–25^. The segregation rates are usually narrower in BC_1_F_1_ as compared to F_2_, while alien transmission rates are higher in disomic lines, especially disomic substitution lines, than monosomic lines^24,25^. Different types (whole-arm or Robertsonian, intercalary and distal) of wheat–*Leymus* translocation lines have also been developed^3,12,26–29^, and Li *et al*.^29^ recorded average translocation frequency of 7.55% for *L. mollis* chromosomes, while Kishii^28^ found that the translocation frequencies of *L. racemosus* chromosomes ranged between 0 and 8%, with higher translocation frequencies in the short arms. However, the unavailability of *L. mollis* genome sequence information and known polymorphic cytological markers to differentiate its chromosomes constitute obstacles to rapid development and adequate characterization of wheat–*L. mollis* chromosome introgression lines (CILs).

*Leymus racemosus*, a close relative of *L. mollis*, has reliable variable cytological markers which enables the application of fluorescence *in situ* hybridization (FISH) to differentiate its chromosomes in wheat–*L. racemosus* addition lines^30^. Using these lines and other wheat–*L. racemosus* CILs for marker validation, we recently developed many *L. racemosus* genome-based DNA markers and successfully transferred a good proportion of the PCR-based markers to *L. mollis* genome^31^. Therefore, in this study, we applied the transferred markers to develop a methodology for producing wheat–*L. mollis* addition lines without *in situ* hybridization. The PCR markers were used for marker-assisted selection, and DArTseq was applied to further genotype the selected lines, allowing development of *L. mollis* chromosome-specific SNP markers to complement the PCR-based markers. In order to validate the marker-based results, genomic *in situ* hybridization (GISH) was applied to confirm the status (disomic or monosomic) of the added chromosomes. The *L. mollis* chromosome-specific markers developed in this study enabled clear differentiation of the wheat–*L. mollis* addition lines, and SNP markers among the two *Leymus* species highlighted their genomic relationship. Also, preliminary phenotypic data summarized the varying effects of the different *L. mollis* chromosomes.

## Methods

### Plant materials

**Figure 1:**
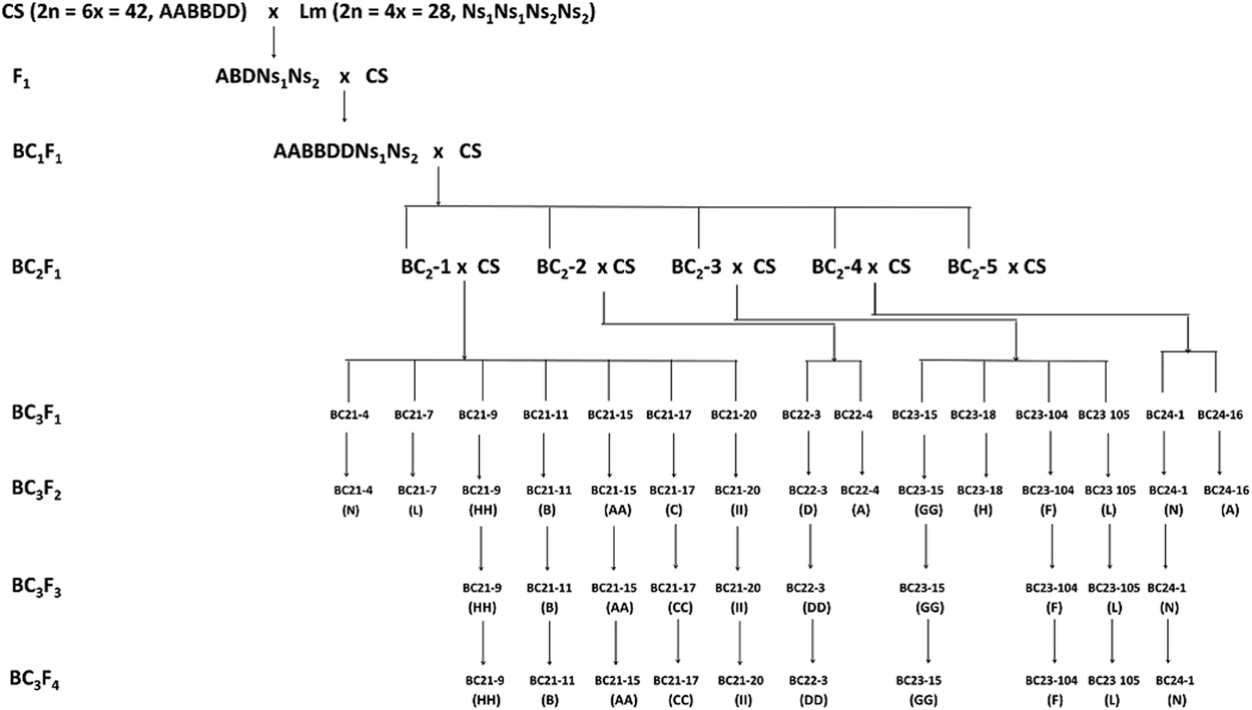
Pedigree of 10 wheat–*L. mollis* chromosome addition lines CS, Chinese Spring; Lm, *L. mollis*; single letters in brackets, monosomic lines; double letters in brackets, disomic lines.

Bread wheat cv. Chinese Spring (CS) (2n = 6x = 42, AABBDD; recipient) was crossed with *L. mollis* (2n = 4x = 28, Ns_1_Ns_1_Ns_2_Ns_2_; donor) to produce wheat–*L. mollis* F_1_ hybrids (2n = 5x = 35; ABDNs_1_Ns_2_). To ensure the survival of the hybrids, embryo rescue was used^32^. The hybrids were backcrossed with CS, and the populations were characterized by chromosome counting and chromosome-specific DNA markers (Fig. 1). The two parents are maintained in the genebank of Tottori Chromosome Bank of Wheat (TACBOW), Arid Land Research Center, Tottori University, with accession numbers KT020-003 (CS) and TACBOW0113 (*L. mollis*). The *L. mollis* accession was originally collected from the Japan seashore (37°5′38″N 140°59′6″E) close to Iwaki, Fukushima, Japan.

### Molecular marker-assisted selection of monosomic and disomic lines

Genomic DNA samples of seedlings of the backcross populations were isolated and purified using the cetyl trimethyl ammonium bromide (CTAB) method and genotyped with *L. racemosus* PCR-based markers transferred to *L. mollis*^31^. Non-carrier segregants and duplicated carriers were discarded, and the remaining plants were advanced to BC_3_F_4_ under a temperature-controlled (22 °C day/18 °C night) greenhouse. In each generation, disomic plants were distinguished using progeny test. In this test, we assumed that monosomic addition lines produce non-carrier (2n = 42, AABBDD), monosomic addition (2n = 43, AABBDD + 1′ [Ns]), and disomic addition (2n = 44, AABBDD + 1′′ [Ns]) segregants, whereas genetically stable disomic plants do not segregate: they produce only disomic addition lines (2n = 44, AABBDD + 1′′ [Ns]). This is because, in meiosis, the monosomic addition lines are expected to produce two different gametes: ABD and ABDNs, while the disomic addition lines should produce one gamete, ABDNs.

### DArTseq genotyping

In order to confirm the presence of *L. mollis* chromosomes in the selected addition lines and develop more *L. mollis* chromosome-specific markers, DArTseq genotyping was applied to rescreen three replicates of each of the lines alongside the two parents and *L. racemosus*. The latter was included to estimate the genomic relationship between the two *Leymus* species because *L. racemosus* genome PCR markers were used to genotype the wheat–*L. mollis* addition lines. All the DNA samples were sent to Diversity Arrays Technology Pty Ltd, Australia (http://www.diversityarrays.com/) for sequencing and marker identification. The genomic representations were sequenced on HiSeq 2500 and the sequences were aligned to wheat_ChineseSpring04 reference and wheat_ConsensusMap_version_4, as our analysis was based on DArTseq platform optimized for hexaploid wheat. DArTseq data in SNP 1 Row Mapping Format, which we used in this study, was scored “0” for a reference allele homozygote (wheat allele only), “1” for an SNP allele homozygote (*L. mollis* allele only), and “2” for a heterozygote (both wheat and *L. mollis* alleles). SNP markers with call rate of 100% (definite scores across all the samples: bread wheat, *L. mollis* and wheat–*L. mollis* genomes) were used for the analysis. Markers with SNP alleles in the wheat genome were discarded. Genetic mapping-related statistics were not considered because our objective was to identify polymorphic markers to differentiate between the wheat, *Leymus* and wheat–*L. mollis* genomes. The data were analyzed for polymorphism between the wheat and *L. mollis* genomes, and polymorphic markers were used to identify *L. mollis* segments in the wheat–*L. mollis* lines. Possible substitutions of wheat chromosomes were analyzed by filtering the SNP markers specific to the *L. mollis* segment (markers with score of “1”) in each line. It should be understood that wheat DArTseq platform grouped all the markers (including *L. mollis*-specific) into the 21 chromosomes of wheat, hence the presence of *L. mollis* chromosome in a wheat–*L. mollis* line would be indicated by a score of “1” if the wheat homoeolog is substituted, or 2 if the homoeolog is not substituted. The former case indicates a substitution line, while the latter indicates an addition line.

On the basis of the correspondence between the SNP alleles and reference alleles in each CS chromosome provided by DArTseq, *L. mollis* chromosome-specific SNP markers were used to determine the homoeologous groups (HGs) of *L. mollis* chromosomes in the wheat background. Genomic relationship between the genomes of the two *Leymus* species was roughly estimated by marker polymorphism between the genomes, whereas markers consistently called (call rate of 100%) among the two genomes, wheat*–L. mollis and* wheat*–L. racemosus* addition lines were used to estimate the relationship between the chromosomes of the two *Leymus* species. This latter set of markers was used for cluster analysis (http://genomes.urv.cat/UPGMA/) to reveal the associations among the chromosomes of the two *Leymus* species.

### Identification of *L. mollis* chromosomes by GISH

*L. mollis* genomic DNA was labeled with fluorescein-12-dUTP (Thermo Scientific) using Random Primers DNA Labeling System (Invitrogen). With the labeled *L. mollis* genomic DNA as probe, GISH was performed for the 10 addition lines following a protocol described for Triticeae species^33^, with slight modifications: steps 3–9 of slide pre-hybridization were skipped and the probe was denatured at 100 °C for 5 min instead of 75 °C for 10 min. After hybridization, the slides were viewed and photographed with an Olympus BX61 automated fluorescence microscope (Olympus).

### Preliminary phenotypic evaluation of the CALs

The 10 addition lines alongside the background wheat cultivar (CS) were laid out in a completely randomized design (CRD) with six replicates in a greenhouse. Seeds of all the genotypes were sown in petri dishes under the same condition, and uniform seedlings of each genotype were transplanted to plastic pots, one plant in each pot. DNA samples of seedlings from monosomic lines were genotyped by PCR to ensure that only alien carriers were transplanted. All the plants grew under a temperature-controlled (22 °C day/18 °C night) condition. Adequate cultural practices necessary for optimum crop performance were observed. Data were taken on number of days to heading and physiological maturity, plant height, spike length, number of spikes per plant, grain yield per spike and grain yield per plant. Two-tailed t-test was applied to compare the mean values of traits between CS and each addition line. At this preliminary stage, differences among the addition lines were not considered, as all the lines are intended to be collectively evaluated under different stress conditions for further selection and production of translocation and recombination lines with desired segments of *L. mollis* chromosomes.

## Results

### Production of wheat–*L. mollis* chromosome addition lines

We developed 10 distinct wheat**–***L. mollis* addition lines (Fig. 1; Table 1). The wheat × *L. mollis* F_1_ hybrids exhibited perennial growth, flowered, but failed to set seed. Repeated attempts to double the chromosomes of the hybrids by colchicine treatment were unsuccessful, so we crossed the hybrids with CS pollens. This approach also failed, but when we crossed in a reciprocal direction, two seeds were harvested. These seeds germinated and grew into fertile annual octoploids (2n = 8x = 56, AABBDDNs_1_Ns_2_). One of them was used to generate backcross populations, from which the addition lines were produced. In the third backcross generation, we selected, by chromosome counting, plants with 43 chromosomes—assumed to be monosomic addition lines—and arbitrarily assigned alphabetical tags (LmA–N) to them (Fig. 1). *Leymus* markers were then used for *L. mollis* chromosome identification and differentiation. Chromosome-specific markers (Table 1) were used to differentiate the lines, while progeny test was used to distinguish between disomic (6) and monosomic (4) lines as we advanced the populations to BC_3_F_4_.

**Table 1:** Identification of wheat–*L. mollis* chromosome addition lines using PCR and DArTseq SNP markers.

Alien chromosome ID	Description	Chromosome constitution (2n)	Total number of markers		Chromosome-specific markers		
			PCR	SNPs	PCR	SNPs	Total
LmA	Disomic addition	21′′ + 1′′	7	810	3	777	780
LmB	Monosomic addition	21′′ + 1′	6	787	1	661	662
LmC	Disomic addition	21′′ + 1′′	7	601	3	500	503
LmD	Disomic addition	21′′ + 1′′	3	454	1	428	429
LmF	Monosomic addition	21′′ + 1′	7	714	2	603	613
LmG	Disomic addition	21′′ + 1′′	6	857	0	757	757
LmH	Disomic addition	21′′ + 1′′	7	757	1	641	642
LmI	Disomic addition	21′′ + 1′′	10	896	3	796	799
LmL	Monosomic addition	21′′ + 1′	5	209	3	185	188
LmN	Monosomic addition	21′′ + 1′	4	620	0	592	592
**All alien chromosomes**	**—**	**—**	**27**	**6317**	**17**	**5940**	**5957**
KT020-003 (CS)	*T*. *aestivum*	42	0	—	—	—	—
TACBOW 0113	*L*. *mollis*	28	47	14530	—	—	—

TACBOW, Tottori Alien Chromosome Bank of Wheat; Lm, *Leymus mollis*; A–N, Lm chromosomes in the wheat genome; ′′, bivalent; ′, univalent; bold, numbers of all the markers that identified Lm chromosomes in the wheat background; CS, Chinese Spring.

### Development of *L. mollis* polymorphic markers and identification of alien chromosomes in the wheat background

From 95 PCR markers transferred from *L. racemosus* to *L. mollis*^31^ and 15,426 SNP markers in the genomes of *L. mollis* and CS, we developed 14,577 *L. mollis* polymorphic markers (47 PCR-based and 14,530 SNP markers) (Table 1 and online Supplementary Table S1). The PCR analysis indicated 49% marker polymorphism between CS and *L. mollis* genomes^31^, whereas the SNP markers revealed a higher polymorphism (~94%) (see online Supplementary Table S1). Chromosomes of *L. mollis* in the wheat background were efficiently identified by 27 PCR markers and 6,317 SNP markers (Table 1). A total of 5,957 *L. mollis* chromosome-specific markers, ranging from 185 in LmL to 796 in LmI (Table 1), enabled unambiguous differentiation of the 10 lines. The number of SNP markers with a genotypic score of 1 (representing only the *L. mollis* allele) in each line ranged between 3 (0.05%) and 15 (0.2%), which lie within genotyping error range. All the lines retained almost 100% of the reference (wheat) alleles in addition to the SNP alleles, showing that none of the CS chromosomes was substituted. Therefore, all the introgressions were confirmed to be addition lines.

### Homoeologous groups of *L. mollis* chromosomes added to wheat

The HGs of all the *L. mollis* chromosomes added to wheat were determined from the correspondence of *L. mollis* chromosome-specific SNP markers to the HGs of CS. The 10 *L. mollis* chromosomes fitted well into seven homoeologous groups, with six chromosomes falling into three HGs (Table 2). Chromosomes in the same HG obviously belong to different sub-genomes, granted that *L. mollis* is a tetraploid species.

**Table 2:** Determination of homoeologous groups of *L. mollis* chromosomes added to the wheat genome.

Alien chromosome ID	Number of SNP markers corresponding to HG of bread wheat (cv. CS) chromosome							HG of Lm chromosome
	1	2	3	4	5	6	7	
LmA	12	**703**	8	15	9	17	13	2
LmB	10	7	6	46	**572**	8	12	5
LmC	16	4	10	14	5	8	**443**	7
LmD	5	6	6	8	5	**385**	13	6
LmF	12	8	**552**	4	9	5	13	3
LmG	6	11	6	36	9	7	**682**	7
LmH	15	11	**585**	5	10	6	9	3
LmI	7	14	10	22	**703**	18	22	5
LmL	**151**	7	4	5	4	6	8	1
LmN	4	9	8	**513**	38	7	13	4

Lm, *L. mollis*; HG, homoeologous group; LmA–N, Lm chromosomes in the wheat genome; bold numbers, number of markers indicating the HG of each chromosome.

### Confirmation of the cytological status of each *L. mollis* chromosome in the addition lines GISH

**Figure 2:**
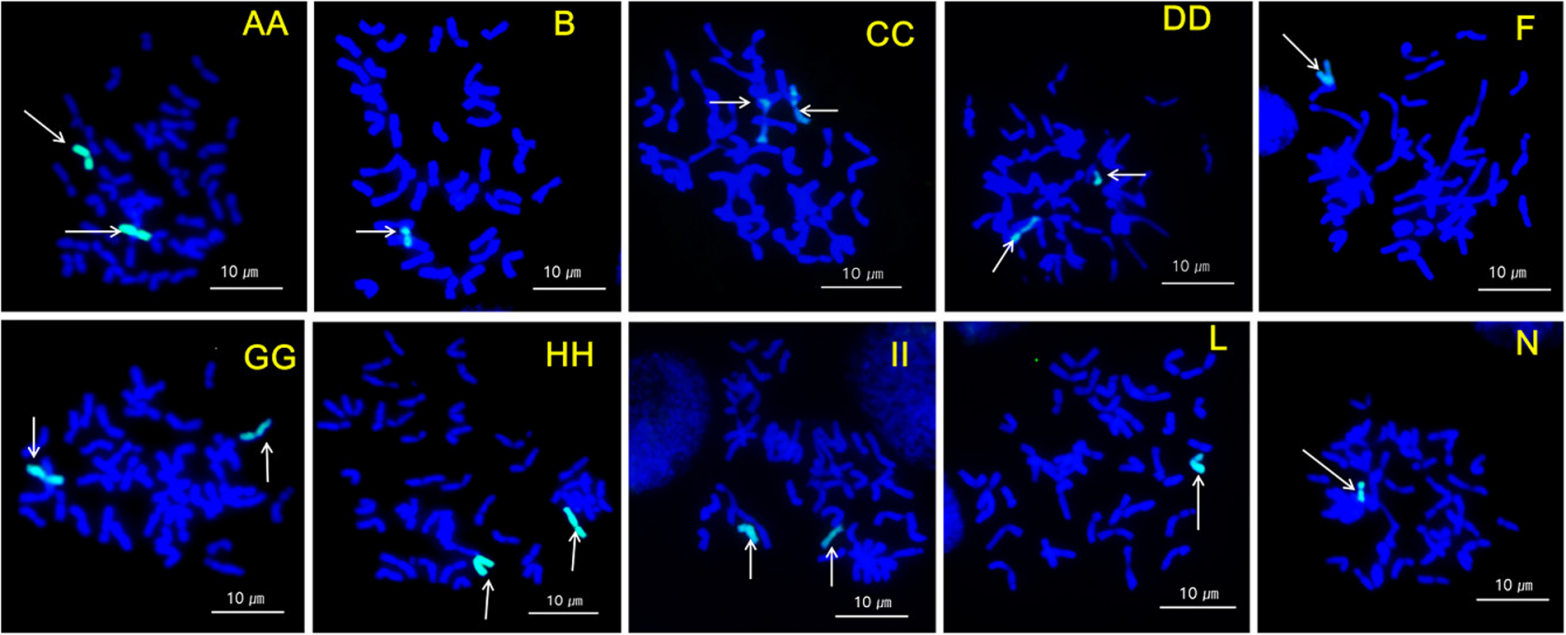
Identification of *L. mollis* chromosomes added to wheat using genomic *in situ* hybridization (GISH). (**A**–**N**), *L. mollis* chromosomes; double letters, disomic lines; single letters, monosomic lines; arrows point to the added chromosomes detected with fluorescein-12-dUTP (green).

Because the use of marker-assisted selection in differentiating monosomic and disomic lines is not a common practice, we validated the status (monosomic or disomic) of *L. mollis* chromosomes in the addition lines by GISH, which confirmed the marker-based results (Fig. 2). All the disomic addition lines were confirmed to have a pair of *L. mollis* chromosomes, whereas the monosomic addition lines had single *L. mollis* chromosomes.

### Relationship between *L. mollis* and *L. racemosus* genomes

**Figure 3:**
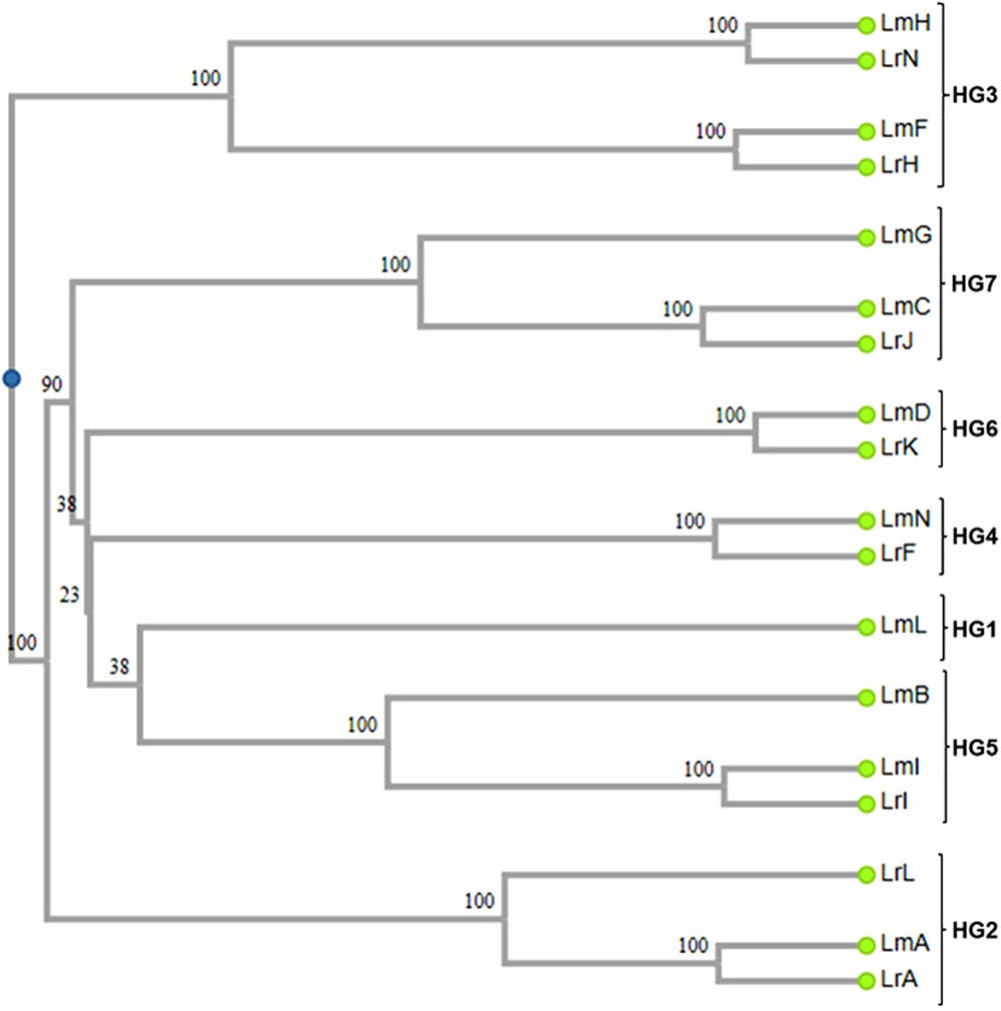
Relationship between *L. mollis* and *L. racemosus* chromosomes LmA–N, *L. mollis* chromosomes; LrA–N, *L. racemosus* chromosomes; numbers at the nodes are bootstrap values; clustering method: Unweighted pair group method with arithmetic mean (UPGMA) (http://genomes.urv.cat/UPGMA/).

Out of 8,653 SNP markers consistently scored in the two *Leymus* genomes, 75% were monomorphic (see online Supplementary Table S2). A cluster analysis using 579 SNP markers among the 10 wheat-*L. mollis* lines produced in this study and 8 wheat–*L. racemosus* addition lines reported previously^30^ revealed an interesting relationship between the chromosomes of the two *Leymus* species. Seven chromosomes each of *L. mollis* and *L. racemosus* in six HGs clustered in pairs: *L. mollis* chromosome H (LmH) and *L. racemosus* chromosome N (LrN); *L. mollis* chromosome F (LmF) and *L. racemosus* chromosome H (LrH); *L. mollis* chromosome C (LmC) and *L. racemosus* chromosome J (LrJ); *L. mollis* chromosome D (LmD) and *L. racemosus* chromosome K (LrK); *L. mollis* chromosome N (LmN) and *L. racemosus* chromosome F (LrF); *L. mollis* chromosome I (LmI) and *L. racemosus* chromosome I (LrI); *L. mollis* chromosome A (LmA) and *L. racemosus* chromosome A (LrA) (Fig. 3). In HG3, *L. mollis* chromosomes LmF and LmH were associated with *L. racemosus* chromosomes LrH and LrN, respectively. Apart from LmL in HG1, whose homoeolog in *L. racemosus* was not produced, every other HG included at least two chromosomes, one from each *Leymus* species. In each HG, the genomic distance between homoeologous chromosomes of the two species was narrower than that within the same species (Table 3). In HG3 for instance, the distance indices between chromosomes LmF and LrH (18) and LmH and LrN (16) are clearly lower than those between LmF and LmH (90) and LrH and LrN (88). Using the genetic association among the chromosomes (Fig. 3; Table 3), we tentatively named the chromosomes (Table 4). For each of the species, we numbered the chromosomes according to their HGs and arbitrarily assigned each chromosome of a homoeologous pair to different sub-genomes, designated as Ns_1_ and Ns_2_; the superscripts “m” and “r” indicate *L. mollis* and *L. racemosus*, respectively. All non-homoeologous chromosomes in each species were assumed to be in the same sub-genome, as homoeology between two chromosomes should not exist in one sub-genome.

**Table 3:** Genomic distance matrix among the chromosomes of *L. mollis* and *L. racemosus*.

	LmA	LmB	LmC	LmD	LmF	LmG	LmH	LmI	LmL	LmN	LrA	LrF	LrH	LrI	LrJ	LrK	LrL	LrN
LmA	0	113	116	112	121	114	120	120	105	110	**20**	110	120	121	120	112	**36**	122
LmB		0	110	103	114	104	115	**78**	103	105	109	105	113	**52**	107	101	106	114
LmC			0	108	116	**78**	117	114	104	106	112	108	116	114	**22**	107	111	117
LmD				0	113	105	114	109	103	106	111	106	113	111	106	**15**	109	115
LmF					0	112	**90**	118	106	112	121	112	**18**	121	120	112	115	**81**
LmG						0	114	111	103	107	112	101	112	112	**43**	103	109	113
LmH							0	124	106	113	121	114	**86**	126	122	115	117	**16**
LmI								0	96	111	117	105	119	**19**	116	108	111	124
LmL									0	103	105	96	101	96	100	97	104	99
LmN										0	106	**21**	110	111	108	107	103	114
LrA											0	111	121	116	116	110	**62**	124
LrF												0	112	108	110	107	108	114
LrH													0	121	120	113	116	**88**
LrI														0	113	111	114	127
LrJ															0	111	113	123
LrK																0	107	116
LrL																	0	119
LrN																		0

LmA–N, *L. mollis* chromosomes; LrA–N, *L. racemosus* chromosomes; emboldened values are distance indices between homoeologous chromosomes. Distance indices were calculated by transforming Pearson’s correlation coefficients (*r*) to distance (d) values using d = (1 − r) × 100 (http://genomes.urv.cat/UPGMA/).

**Table 4:** Proposed nomenclature system for *Leymus* chromosomes.

S/N	*L. mollis* chromosomes				*L. racemosus* chromosomes			
	Arbitrary ID	Status in wheat background	HG	Proposed name	Arbitrary ID	Status in wheat background	HG	Proposed name
1	LmL	Monosomic	1	1Ns_1_^**m**^	—	—	1	—
2	—	—	1	—	—	—	1	—
3	LmA	Disomic	2	2Ns_1_^**m**^	LrA	Disomic	2	2Ns_1_^**r**^
4	—	—	—	—	LrL	Disomic	2	2Ns_2_^**r**^
5	LmF	Monosomic	3	3Ns_1_^m^	LrH	Disomic	3	3Ns_1_^r^
6	LmH	Disomic	3	3Ns_2_^m^	LrN	Disomic	3	3Ns_2_^r^
7	LmN	Monosomic	4	4Ns_1_^**m**^	LrF	Disomic	4	4Ns_1_^**r**^
8	—	—	4	—	—	—	4	—
9	LmI	Disomic	5	5Ns_1_^**m**^	LrI	Disomic	5	5Ns_1_^**r**^
10	LmB	Monosomic	5	5Ns_2_^**m**^	—	—	5	—
11	LmD	Disomic	6	6Ns_1_^**m**^	LrK	Disomic	6	6Ns_1_^**r**^
12	—	—	6	—	—	—	6	—
13	LmC	Disomic	7	7Ns_1_^**m**^	LrJ	Disomic	7	7Ns_1_^**r**^
14	LmG	Disomic	7	7Ns_2_^**m**^	—	—	7	—

HG, homoeologous group; Lm, *Leymus mollis*; Lr, *L. racemosus*; Ns_1_ and Ns_1_, different Ns genomes^9^; superscript **m** and **r**, Ns genomes in *L. mollis* and *L. racemosus*, respectively; A–N, *Leymus* chromosomes in the wheat genome; —, chromosomes either not identified or whose HGs have not been clearly established by our analysis.

### Phenotypic variation between CS and the addition lines

**Figure 4:**
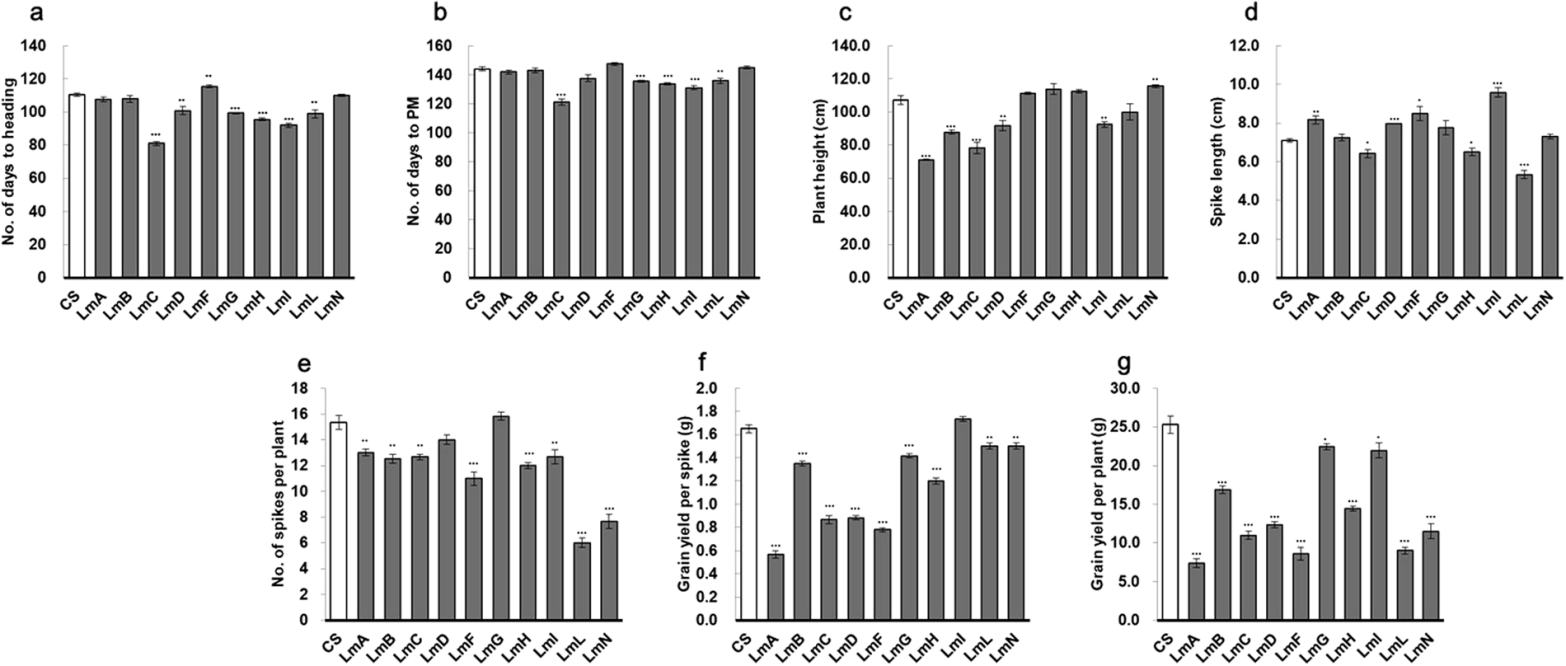
Variation in quantitative traits between wheat (cv. CS) and wheat-*L. mollis* addition lines. CS, Chinese Spring; LmA–N, *L. mollis* chromosomes; PM, physiological maturity; *, p < 0.05; **, p < 0.01; ***, p < 0.001; error bars, standard errors of the means. Mean values (n = 6) between CS and each addition line were compared using two-tailed t-test. Differences among the lines were not considered at this preliminary stage, as all the lines will be subjected to different conditions of stress for further selection and production of translocation and recombination lines with desired segments of *L. mollis* chromosomes.

From the results of the preliminary evaluation we conducted (Fig. 4; Supplementary Table S3), all the addition lines were significantly different from CS in at least one of the seven traits measured, indicating the effects of *L. mollis* chromosomes in the lines. Number of days to heading (DH) was significantly reduced in six of the lines, significantly increased in one, while three lines were not significantly different from CS (Fig. 4a). Five of the six lines with significantly reduced DH also reached physiological maturity significantly earlier than CS, while the other five lines were not significantly different from CS (Fig. 4b). Plant height was significantly reduced in five lines, significantly increased in one line, while four lines were not different from CS (Fig. 4c). All the lines were significantly different from CS in at least one yield component (Fig. 4d–g). Although spikes were significantly longer in some of the lines (Fig. 4d), number of spikes per plant, grain yield per spike and grain yield per plant were significantly lower in all the lines, except LmG and LmI, which were not significantly different from CS in number of spikes per plant and grain yield per spike, respectively.

## Discussion

In our experience^30,34^, production of wheat–alien CILs from intergeneric F_1_ hybrid plants requires at least eight years of laborious cytogenetic and/or phenotypic screening. Here, we have shown that marker-assisted selection can considerably reduce the time and effort. In this study, marker development, genotyping, selection and confirmation of the status of each alien chromosome in the 10 addition lines were completed within three years. Our marker-based approach of identifying disomic lines was supported by GISH results. Therefore, the use of molecular markers from the genomes of important wild relatives to track their chromosomes in wheat is a reliable method to facilitate speedy expansion of diversity in wheat germplasm^31,35,36^. This approach would incentivize wheat breeders to develop cultivars with improved adaptability to keep pace with the projected global increase in wheat demand^37^.

We avoided spurious duplication of different segments of the same chromosome in different addition lines. The chromosome-specific markers developed in this study clearly differentiated the 10 lines. The duplicates we initially selected by chromosome counting were clarified by molecular markers. Identical PCR amplifications and DArTseq genotypic data revealed lines carrying the same *L. mollis* chromosome. This would not be possible with GISH because GISH uses genomic DNA as probe, which can only identify the alien chromosomes but cannot show differences between them. Using phenotypic data to identify plants that carry different segments of the same chromosome is difficult; hence, they may be mistaken as carriers of different chromosomes. Such situations can be effectively managed with appropriate molecular markers.

Determination of the HG of each *L. mollis* chromosome using the chromosome-specific SNP markers was consistent with the chromosome clustering using shared SNP markers. The HGs of the remaining *L. mollis* chromosomes, if introgressed in wheat, can be easily determined with this approach. Other methods of HG determination can result in assignment of different HGs to the same chromosome^30,38^. To determine the HGs of alien chromosomes, their ability to substitute wheat chromosomes in interspecific or intergeneric hybrids and functionally compensate the substituted chromosomes in substitution lines is usually applied^39–42^. This approach is difficult and its accuracy relies, to a large extent, on the morphological similarities between the substitution line and the wild type, which is under environmental influences.

The high proportion of monomorphic markers in the two genomes of *Leymus* species strongly indicates high genomic similarity between the genomes, and the association of their chromosomes suggests that the genomic distance between the sub-genomes of the two species is smaller than the distance between the sub-genomes within each of the species. This gives the impression that the two *Leymus* species may have evolved from independent hybridization events of the same diploid species with partially differentiated genomes, for instance Ns_1_ and Ns_2_. However, at the moment, we cannot draw a convincing conclusion on the evolutionary relationship between the two *Leymus* species or their sub-genomes, as this would require the application of the genomic resources of their diploid progenitors as analyzers of the polyploid genomes. Diploid analyzers would also enable reliable discrimination of the sub-genomes and help to clarify the sources of the elementary genomes of the *Leymus* species. Noteworthy is that the chromosomes of hybrids from *Leymus* species form complete meiotic pairs^43–46^, which is a rare phenomenon in interspecific hybrids. This and the high transferability of DNA markers between *L. racemosus* and *L. mollis* suggest that the genomic resources of any of the two species should be interchangeably deployed to analyze their genomes. But we could not characterize *L. mollis* chromosomes with *L. racemosus* cytological markers, so the genomic difference between these species still needs to be clarified. The tentative nomenclature for *Leymus* chromosomes proposed here can easily accommodate all the chromosomes of *Leymus* species, irrespective of ploidy level. Considering the consistency of HGs determined independently using the chromosome-specific and common SNP markers, we are certain about the HGs of the *L. mollis* chromosomes, but the accuracy of the assignment of the chromosomes to the sub-genomes needs to be confirmed using diploid analyzers.

Interestingly, the preliminary phenotypic results obtained in this study have shown that *L. mollis* chromosome segments can be used to develop early maturing cultivars which can be cultivated in areas with short periods of favourable wheat growing conditions. However, the exact segment of the chromosome(s) should be identified and used to produce translocation or recombination lines, to reduce the effects of deleterious genes which may have caused the reduction in the yield components observed in the addition lines. At the moment, since all the genotypes were evaluated under normal growth conditions, we cannot conclude that the relative superior yield components of CS will be sustained under stress conditions. Therefore, all the genotypes will be evaluated under different simulated and actual stress conditions to explore the possibility of selecting lines with the sturdy traits of *Leymus* species^6,10–13^.

Essentially, we have proven that wheat–alien addition lines can be rapidly developed and reliably characterized using DNA markers for effective selection of carriers and recognition of disomic and monosomic lines. This approach requires the development of chromosome-specific DNA markers from the genomes of potential gene donors or closely related sequenced genomes. The integration of DArTseq genotyping allows confirmation of the PCR results, development of more chromosome-specific markers, and further characterization of the addition lines. Allocation of all the markers on the DArTseq platform to wheat chromosomes enables effective analysis of wheat–alien complexes. The application of genotyping-by-sequencing approaches, including DArTseq, in the analysis of germplasm of wheat and other plants has gained reasonable popularity^47–50^. This is not the case with the characterization of wheat-alien introgression lines involving distant relatives of wheat, possibly because it may be thought that wide genetic distance and differences in the ploidy levels of bread wheat and these relatives would not allow identification of homoeology between their genomes. Our previous report on wheat–*L. racemosus*^31^ and the results of this study have shown clearly that alien segment-specific markers can easily be isolated and the correspondence of alien chromosome-specific SNP markers with bread wheat reference alleles can be used to determine the HGs of the alien chromosomes. This study has clearly demonstrated that DArTseq SNPs can be integrated with PCR markers to produce and characterize wheat–alien addition lines without necessarily applying *in situ* hybridization for alien chromosome identification. Given its reliability and savings in time and efforts, we recommend the use of this simple methodology to accelerate introgression breeding of wheat.

## Electronic supplementary material

Table S1

Table S2

Table S3

## References

[1] Anamthawat-Jonsson K (2001). Genetic and genomic relationships in *Leymus* Hochst. Hereditas.

[2] Anamthawat-Jonsson K (2009). Evolutionary diversification of satellite DNA sequences from *Leymus* (Poaceae: Triticeae). Genome.

[3] Chen P (2005). Development and characterization of wheat–*Leymus racemosus* translocation lines with resistance to *Fusarium* Head Blight. Theor Appl Genet.

[4] Fan X (2014). Evolutionary pattern of rDNA following polyploidy in *Leymus* (Triticeae: Poaceae). Mol Phylogenet Evol.

[5] Fan X (2009). Phylogeny and evolutionary history of Leymus (Triticeae; Poaceae) based on a single-copy nuclear gene encoding plastid acetyl-CoA carboxylase. BMC Evol Biol.

[6] Yang X (2015). Development and molecular cytogenetic identification of a novel wheat–*Leymus mollis* Lm#7Ns (7D) disomic substitution line with stripe rust resistance. PLoS One.

[7] Zhang H, Dvorak J (1991). The genome origin of tetraploid species of *Leymus* (Poaceae: Triticeae) inferred from variation in repeated nucleotide sequences. Am J Bot.

[8] Wang RR, Jensen KB (1994). Absence of the J genome in *Leymus* species (Poaceae: Triticeae): evidence from DNA hybridization and meiotic pairing. Genome.

[9] Anamthawat-Jonsson K (2014). Molecular cytogenetics of *Leymus*: Mapping the Ns genome-specific repetitive sequences. J Syst Evol.

[10] McGuire P, Dvorak J (1981). High salt-tolerance potential in wheatgrasses. Crop Science.

[11] Xiao X, Sha L, Fan X, Zhou Y (2012). Salt tolerance on seed germination of five *Leymus* Species. World Journal of Agricultural Sciences.

[12] Bao Y, Wang J, He F, Ma H, Wang H (2012). Molecular cytogenetic identification of a wheat (Triticum aestivum) –American dune grass (*Leymus mollis*) translocation line resistant to stripe rust. Genet Mol Res.

[13] Zhang A (2017). Molecular cytogenetics identification of a wheat–*Leymus mollis* double disomic addition line with stripe rust resistance. Genome.

[14] Wang L, Chen P (2008). Development of Triticum aestivum–*Leymus racemosus* ditelosomic substitution line 7Lr#1S(7A) with resistance to wheat scab and its meiotic behavior analysis. Chin Sci Bull.

[15] Qi LL, Pumphrey MO, Friebe B, Chen PD, Gill BS (2008). Molecular cytogenetic characterization of alien introgressions with gene Fhb3 for resistance to Fusarium head blight disease of wheat. Theor Appl Genet.

[16] Yang XF (2014). Chromosome constitution and origin analysis in three derivatives of Triticum aestivum–*Leymus mollis* by molecular cytogenetic identification. Genome.

[17] Yang XF (2017). Isolation and molecular cytogenetic characterization of a wheat–*Leymus mollis* double monosomic addition line and its progenies with resistance to stripe rust. Genome.

[18] Mohammed YSA (2014). Impact of wheat–*Leymus racemosus* added chromosomes on wheat adaptation and tolerance to heat stress. Breeding Sci.

[19] Mohammed YS, Eltayeb AE, Tsujimoto H (2013). Enhancement of aluminum tolerance in wheat by addition of chromosomes from the wild relative *Leymus racemosus*. Breed Sci.

[20] Liu X, Shi J, Zhang XY, Ma YS, Jia JZ (2001). Screening salt tolerance germplasms and tagging the tolerance gene(s) using microsatellite (SSR) markers in wheat. Acta Bot Sin.

[21] Tsitsin NV (1965). Remote hybridization as a method of creating new species and varieties of plants. Euphytica.

[22] Wang J (2013). Morphological and molecular cytogenetic characterization of partial octoploid. Tritileymus. Genet Resour Crop Ev.

[23] Fu J, Chen S, Zhang A (1993). Studies of the formation and cytogenetics of octoploid *Tritileymus*. Acta Genet Sin.

[24] Fu J, Chen S, Zhang A, Hou W, Yang Q (1996). Cytogenetic studies on the cross progenies between octoploid *Tritileymus* and *Triticum aestivum*. Acta Genet Sin.

[25] Fu J (1997). Cytogenetic studies on the cross between octoploid *Tritileymus* and nullisomic wheat. Acta Genet Sin.

[26] Pang YH (2014). Molecular cytogenetic characterization of a wheat–*Leymus mollis* 3D(3Ns) substitution line with resistance to leaf rust. J Genet Genomics.

[27] Pang YH (2014). Cytogenetic and molecular identification of a wheat–*Leymus mollis* alien multiple substitution line from octoploid *Tritileymus* x Triticum durum. Genet Mol Res.

[28] Kishii M (2011). Production of wheat–*Leymus racemosus* translocation lines. eWIS.

[29] Li H (2015). Development of *Triticum aestivum*–*Leymus mollis* translocation lines and identification of resistance to stripe rust. J Genet Genomics.

[30] Kishii M, Yamada T, Sasakuma T, Tsujimoto H (2004). Production of wheat–*Leymus racemosus* chromosome addition lines. Theor Appl Genet.

[31] Edet OU (2018). Efficient anchoring of alien chromosome segments introgressed into bread wheat by new *Leymus racemosus* genome-based markers. BMC Genet.

[32] Cisneros A, Tel-Zur N (2010). Embryo rescue and plant regeneration following interspecific crosses in the genus Hylocereus (Cactaceae). Euphytica.

[33] Brammer, S. P., Vasconcelos, S., Poersch, L. B., Oliveira, A. R. & Brasileiro-Vidal, A. C. Genomic *in situ* hybridization in Triticeae: A methodological approach. In: *Plant Breeding from Laboratories to* Fields (ed Andersen S. B.) 1–22 (InTech, Rijeka, 2013).

[34] Kishii M (2010). Production of wheat–*Psathyrostachys huashanica* chromosome addition lines. Genes Genet Syst.

[35] Zhang J (2017). A resource of large-scale molecular markers for monitoring *Agropyron cristatum* chromatin introgression in wheat background based on transcriptome sequences. Sci Rep.

[36] Gong W (2017). Agronomic traits and molecular marker identification of Wheat–*Aegilops caudata* addition lines. Front Plant Sci.

[37] Shiferaw B (2013). Crops that feed the world 10. Past successes and future challenges to the role played by wheat in global food security. Food Secur.

[38] Larson SR (2012). *Leymus* EST linkage maps identify 4NsL-5NsL reciprocal translocation, wheat–*Leymus* chromosome introgressions, and functionally important gene loci. Theor Appl Genet.

[39] Morris, R. & Sears, E. R. The cytogenetics of wheat and its relatives. In: *Wheat and wheat improvement* (eds Quisenberry, R. S. & Reitz, L. P.) 19–87 (Madison, 1967).

[40] Molnar-Lang, M., Molnar, I., Szakacs, E., Linc, G. & Bedo, Z. Production and molecular cytogenetic identification of wheat-alien hybrids and introgression lines. In: *Genomics of plant genetic resources: managing sequencing and mining genetic resources* Vol. 1 (eds Tuberosa, R., Graner, A. & Frison, E.) 255–284 (Springer, 2014).

[41] Calderon MD, Ramirez MD, Martin A, Prieto P (2012). Development of *Hordeum chilense* 4Hch introgression lines in durum wheat: a tool for breeders and complex trait analysis. Plant Breeding.

[42] Badaeva ED, Budashkina EB, Badaev NS, Kalinina NP, Shkutina FM (1991). General features of chromosome substitutions in *Triticum aestivum* x *T. timopheevii* hybrids. Theor Appl Genet.

[43] Dewey D (1972). Cytogenetics of tetraploid *Elymus cinereus*, *E. triticoides*, *E. multicaulis*, *E. karatviensis*, and their F1 hybrids. Bot Gaz.

[44] Dewey D (1970). Genome relations among diploid *Elymus junceus* and certain tetraploid and octoploid *Elymus* species. Am J Bot.

[45] Dewey, D. R. The genomic system of classification as a guide to intergeneric hybridization with the perennial Triticeae. In: *Gene manipulation in plant improvement* (ed Gustafson, J. P.) 209–279 (Plenum Publishing Corporation, 1984).

[46] Kishii M, Wang RR, Tsujimoto H (2003). Characteristics and behaviour of the chromosomes of *Leymus mollis* and *L. racemosus* (Triticeae, Poaceae) during mitosis and meiosis. Chromosome Res.

[47] Li H (2015). A high density GBS map of bread wheat and its application for dissecting complex disease resistance traits. BMC Genomics.

[48] Baloch, F. S. *et al*. A whole genome DArTseq and SNP analysis for genetic diversity assessment in durum wheat from Central Fertile Crescent. *Plos One* **12**, doi:ARTN e016782110.1371/journal.pone.0167821 (2017). 10.1371/journal.pone.0167821 PMC5242537 28099442

[49] Davey JW (2011). Genome-wide genetic marker discovery and genotyping using next-generation sequencing. Nat Rev Genet.

[50] Andrews KR, Good JM, Miller MR, Luikart G, Hohenlohe PA (2016). Harnessing the power of RADseq for ecological and evolutionary genomics. Nat Rev Genet.

