# Supplementary material for: Novel molecular marker-assisted strategy for production of wheat–Leymus mollis chromosome addition lines
Source: Sci Rep. 2018 Oct 31;8:16117. doi: 10.1038/s41598-018-34545-x (PMC6208378; doi:10.1038/s41598-018-34545-x)
Supplement: Supplementary file 3 — Table S3 [file 41598_2018_34545_MOESM3_ESM.pdf]

Novel molecular marker-assisted strategy for production of wheat–*Leymus mollis* chromosome addition lines

Offiong U. Edet[1,2], Yasir S. A. Gorafi[1,3], Seong-woo Cho[4], Masahiro Kishii[5] and

*Hisashi Tsujimoto[1] (tsujim@alrc.tottori-u.ac.jp)

[1]Arid Land Research Center, Tottori University, Tottori, Japan

[2]United Graduate School of Agricultural Sciences, Tottori University, Tottori, Japan

[3]Agricultural Research Corporation (ARC), Wad Madani, Sudan

[4]Department of Crop Science and Biotechnology, Chonbuk National University, Jeonju, Republic of Korea

[5]International Maize and Wheat Improvement Center (CIMMYT), El Batan, Mexico

Table S3 T-test P-values of mean difference in traits between wheat (CS) and 10 wheat-*L. mollis* chromosome addition lines

Genotype ID	No. of days to heading	No. of days to PM	Plant height (cm)	Spike length (cm)	No. of spikes per plant	Grain yield per spike	Grain yield per plant
LmA	0.1352	0.3025	0.0000	0.0024	0.0067	0.0000	0.0000
LmB	0.3267	0.6253	0.0003	0.4086	0.0023	0.0000	0.0003
LmC	0.0000	0.0000	0.0000	0.0192	0.0036	0.0000	0.0000
LmD	0.0094	0.0501	0.0032	0.0000	0.0780	0.0000	0.0000
LmF	0.0026	0.0546	0.1940	0.0107	0.0002	0.0000	0.0000
LmG	0.0000	0.0004	0.1357	0.1249	0.4556	0.0004	0.0494
LmH	0.0000	0.0001	0.1147	0.0228	0.0010	0.0000	0.0001
LmI	0.0000	0.0000	0.0012	0.0000	0.0070	0.0702	0.0486
LmL	0.0054	0.0065	0.2318	0.0002	0.0000	0.0064	0.0000
LmN	0.7669	0.5353	0.0219	0.2197	0.0000	0.0064	0.0000

PM, physiological maturity; LmA–N, *L. mollis* chromosomes
